# Distinctive Patterns of Initially Presenting Metastases and Clinical Outcomes According to the Histological Subtypes in Stage IV Non-Small Cell Lung Cancer

**DOI:** 10.1097/MD.0000000000002795

**Published:** 2016-02-12

**Authors:** Dong Soo Lee, Yeon S. Kim, Chul S. Kay, Sung H. Kim, Chang D. Yeo, Jin W. Kim, Seung Joon Kim, Young K. Kim, Yoon H. Ko, Jin H. Kang, Kyo Y. Lee

**Affiliations:** From the Department of Radiation Oncology (DSL, YSK, CSK, SHK); Division of Pulmonology (CDY, JWK, SJK, YKK), Department of Internal Medicine; Division of Medical Oncology (YHK, JHK), Department of Internal Medicine; Department of Hospital Pathology (KYL); and The Cancer Research Institute (SJK), College of Medicine, The Catholic University of Korea, Seocho-gu, Seoul, South Korea.

## Abstract

This study was designed to compare the primary patterns of metastases and clinical outcomes between adenocarcinoma (Adenoca) and squamous cell carcinoma (SQ) in initially diagnosed stage IV non-small cell lung cancer (NSCLC).

Between June 2007 and June 2013, a total of 427 eligible patients were analyzed. These patients were histologically confirmed as Adenoca or SQ and underwent systemic imaging studies, including 18F-fluorodeoxyglucose positron emission tomography/computed tomography and brain imaging. Synchronous metastatic sites were categorized into 7 areas, and whole-body metastatic scores were calculated from 1 to 7 by summation of each involved region. We compared the patient, tumor, and metastatic characteristics according to the histological subtypes, and examined clinical outcomes.

The enrolled study cohort comprised 81% (n = 346) Adenoca patients and 19% (n = 81) SQ patients. The median age of the study population was 65 years (range, 30–94 years), and 263 (61.6%) patients were male. The most common metastatic sites were thoracic lymph nodes (LNs) (84.3%), followed by lung to lung/lymphangitic spread (59%) and bone (54.8%). The distribution of patient characteristics revealed that age ≥65 years (69.1% vs 50.6%; *P* = 0.003) and male sex (84% vs 56.4%; *P* < 0.001) were more frequently found in SQ patients. Regarding metastatic features, bone metastasis (60.4% vs 30.9%; *P* < 0.001), lung to lung/lymphangitic metastasis (63% vs 42%; *P* = 0.001), and brain metastasis (35% vs 16%; *P* = 0.001) were significantly and more frequently found in Adenoca patients. Patients with high metastatic scores (score 3–6) were more frequently found to have Adenoca (91.6% vs 73.4%; *P* < 0.001). In multivariate prognostic evaluation, sex (*P* = 0.001), age (*P* < 0.001), histology (*P* < 0.001), LN status (*P* = 0.032), pleural/pericardial metastasis (*P* = 0.003), abdomen/pelvis metastasis (*P* < 0.001), axilla/neck metastasis (*P* = 0.006), and treatment factors (*P* < 0.001) remained independent prognostic factors affecting overall survival.

We observed distinctive patterns of primary metastases and clinical outcomes according to the histological subtypes in stage IV NSCLC. Future studies need to disclose the underlying mechanism of these unique metastatic features and tumor biologies.

## INTRODUCTION

Lung cancer, usually detected at an advanced stage, has a dismal 5-year survival rate of only 15%,^1^ and remains the leading cause of cancer-related deaths worldwide in both males and females.^2^ Among males, lung cancer was the most frequently diagnosed cancer and the leading cause of cancer death in 2012 global cancer statistics.^2^ Among females, lung cancer was the foremost cause of cancer death in more developed countries, and the second leading cause of cancer death in less developed countries.^2^ According to recent cancer statistics in South Korea, the incidences of lung cancer have decreased in males and have increased in females.^3^ There were 15,167 males and 6,586 females reported as new cases in 2011, and the age-standardized risks of mortality due to lung cancer have decreased slightly in both sexes since 2002.^3^

The universal lung cancer epidemiology has been continuously changing, and nonsquamous non-small cell lung cancer (NSCLC) in never-smoking women has been gradually increasing.^4,5^ In a large US population-based study, lung cancer age-adjusted incidence rates were significantly lower in never smokers than former or current smokers. Adenocarcinoma (Adenoca) was more common in never smokers than in former or current smokers.^6^

A number of factors have been addressed in the prognosis about NSCLC.^7–15^ Epidermal growth factor receptor (EGFR) is a 170-kDa transmembrane receptor tyrosine kinase with an intracellular regulatory domain with tyrosine kinase activity and an extracellular ligand-binding domain.^16^ EGFR gene mutations are frequently (30%–50%) found in lung Adenoca as well as in Asian women who are nonsmokers and are the most important decisive factors for tyrosine kinase inhibitor (TKI) treatment in advanced lung Adenoca.^17^ Although the carcinogenic etiology and natural history in squamous cell carcinoma (SQ) and Adenoca, the 2 most common subtypes of NSCLC, are completely individualized, the unique process of metastatic spread and prognostic disparities is not wholly clarified. Thus, we undertook the present study to determine whether there are distinctive patterns of metastases and different clinical outcomes according to histological types in a primarily diagnosed stage IV NSCLC population.

## MATERIALS AND METHODS

### Study Population

We retrospectively reviewed the electronic medical records of newly diagnosed stage IV NSCLC patients who were registered in our hospital database between June 2007 and June 2013. The following criteria were used for study enrollment: patients who were initially diagnosed with stage IV lung Adenoca or SQ by histopathology; patients who underwent whole-body imaging studies, including 18F-fluorodeoxyglucose positron emission tomography/computed tomography (18F-FDG-PET/CT) and brain imaging; and patients who had accessible survival information. Patients with pathological subtypes other than Adenoca or SQ and patients who did not undergo pretreatment whole-body imaging studies were excluded from this study. Finally, a total of 427 patients met the entire criteria and were included in the present study. The Institutional Review Board of the Catholic Medical Center Ethics Committee approved this retrospective cohort study.

### Evaluation of Patients With Metastatic Characteristics

The patient demographics and tumor characteristics were analyzed. In terms of whole-body metastatic evaluation, the formerly developed whole-body metastatic score was utilized. Briefly, we measured the metastatic extent based on metabolic imaging using 18F-FDG-PET/CT. We also referred to the CT of the whole chest/abdomen, and magnetic resonance imaging (MRI) or CT of the brain. For evaluation of central nervous system metastasis, MRI of the brain was preferentially performed. Cytologic assessment of pleural or pericardial effusion was conducted when clinically indicated. Synchronous metastatic sites were categorized into 7 areas with the following: abdomen/pelvis (including liver, adrenal gland, lymph nodes [LNs], or other abdomino-pelvic organs); lung to lung or pulmonary lymphangitic spread; bone (skeletal system); pleural and/or pericardial metastasis; upper neck and/or axillary LNs; other soft tissue; and brain. Total scores were calculated from 1 to 7 by summation of each score as previously described.^13,14^ Thoracic LN status was also measured as cN0 or cN+ (metabolically positive cN1, cN2, or cN3 stations). Other detailed explanations for metastatic estimation are described in our prior studies.^13,14^

In terms of treatment factors, the administered types of treatment were categorized into systemic chemotherapy/targeted therapy or no treatment/follow-up loss at our institution.

### Mutational Analysis of EGFR

The EGFR mutational status was retrospectively investigated from the NSCLC patients. Activating EGFR mutations from exons 18 to 21 were analyzed using a direct sequencing method from formalin-fixed paraffin-embedded lung tumor tissues. The detailed methods of EGFR mutation testing are described in our previous study.^12^ The survival of patients who were treated with EGFR-TKI was also analyzed.

### Survival Assessment

Overall survival (OS) was calculated from the date of the initial diagnosis to the date of death or last follow-up. There were some patients who were lost in follow-up at our institution. Therefore, we acquired the survival data from the Korean Central Cancer Registry. Using this method, we could access the final status of patients in the included population.

### Statistical Analyses

Statistical analyses were carried out using SPSS software (ver. 12.0; SPSS Inc, Chicago, IL). Pearson χ^2^ test or Fisher exact test was conducted to evaluate the differences between groups for categorical variables. The Kaplan-Meier method was used to generate survival curves, and comparison between curves was performed using the log-rank test. Cox proportional hazards model was utilized to identify independent factors in multivariate survival analysis. All statistical results were 2-sided and were considered statistically significant at a *P* < 0.05.

## RESULTS

### Patient, Tumor, and Metastatic Characteristics

The patient and tumor characteristics are shown in Table 1. The median age was 65 years (range, 30–94 years), and 263 (61.6%) patients were male. The proportions of never and ever smokers were 49.1% (n = 170) and 45.1% (n = 156), respectively, among 346 Adenoca patients, and 17.3% (n = 14) and 76.5% (n = 62), respectively, among 81 SQ patients. In terms of histological subtype, Adenoca and SQ comprised 81% and 19%, respectively. Among the 346 Adenoca patients, EGFR mutation testing was performed in 249 (71.9%), and 98 (39.4%) patients exhibited activating EGFR mutations. The detailed distribution of EGFR mutations in the 98 patients was as follows: exon 19 mutation in 48 (49%); exon 20 mutation in 6 (6.1%); exon 21 mutation, including Leu858Arg mutation, in 42 (42.9%); and double mutations in 2 (2%) patients. Table 2 shows the description of the metastatic characteristics. The most common metastatic sites were LNs followed by lung to lung/lymphangitic metastasis and bone.

**TABLE 1:**
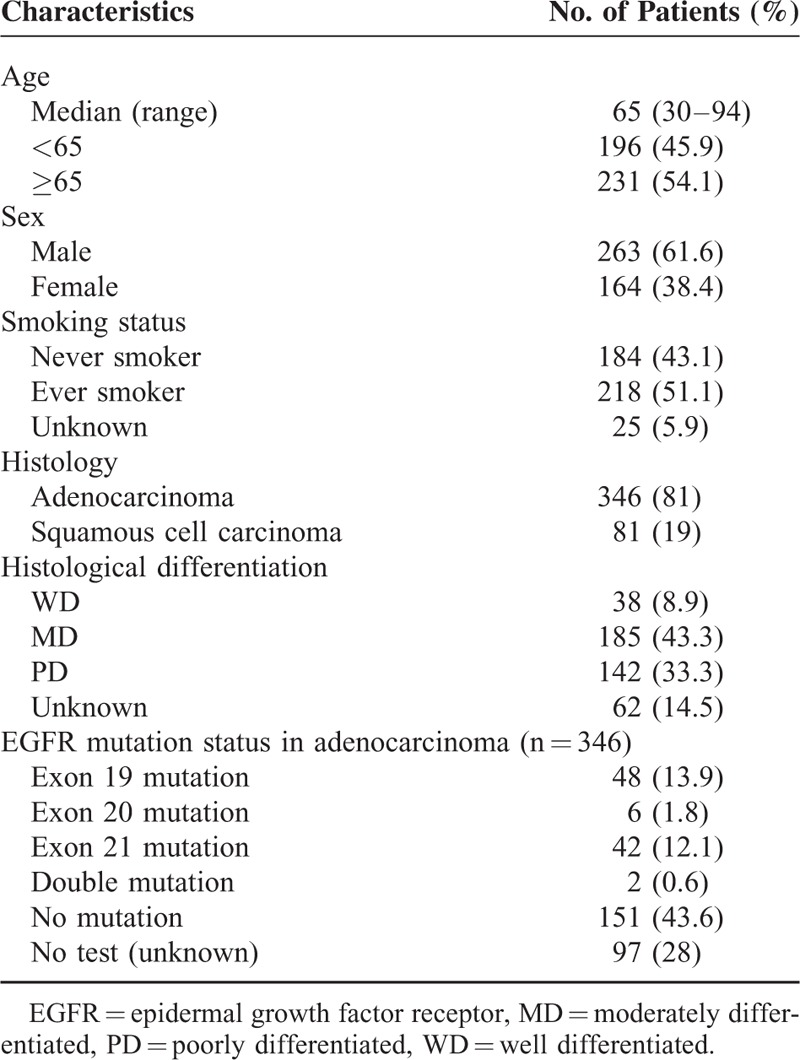
The Patient and Tumor Characteristics

**TABLE 2:**
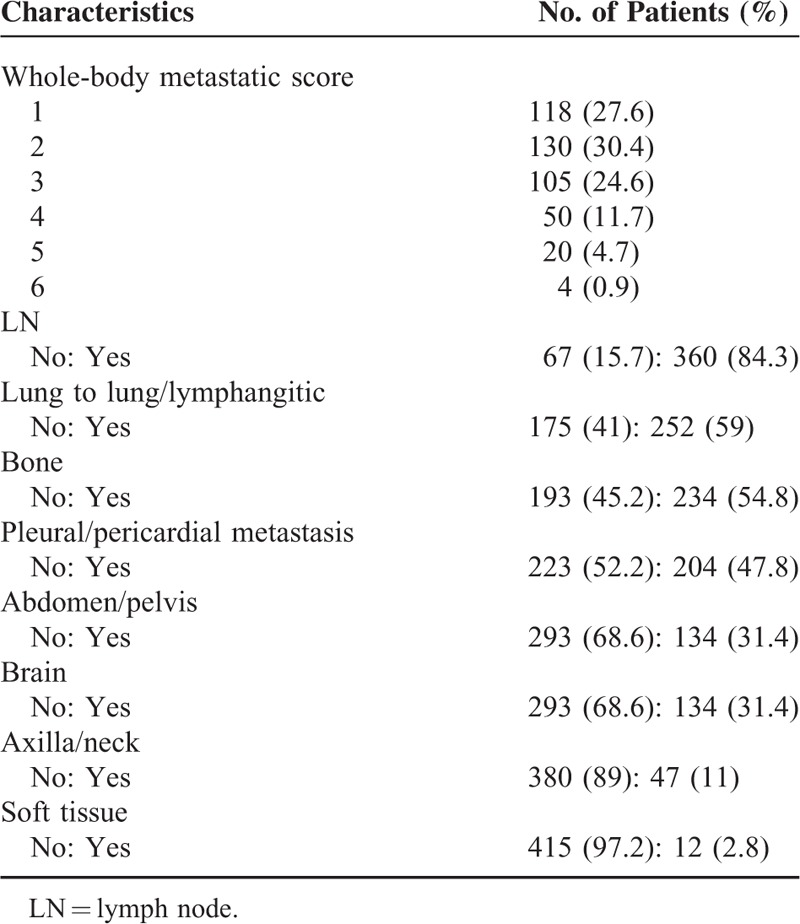
Metastatic Characteristics in the Entire Population

### Comparison of Tumor and Metastatic Characteristics According to Histological Subtype

We compared in detail the distribution of patient, tumor, and metastatic characteristics between Adenoca and SQ (Table 3). Advanced age (≥65 years) and male sex were more frequently found in SQ. Although the proportion of LN status was not different between the histological subtypes (*P* = 0.661), lung to lung/lymphangitic metastasis (63% vs 42%; *P* = 0.001), bone metastasis (60.4% vs 30.9%; *P* < 0.001), and brain metastasis (35% vs 16%; *P* = 0.001) were significantly and more frequently found in Adenoca. By contrast, abdomen/pelvis metastasis (40.7% vs 29.2%; *P* = 0.044) was more frequently found in SQ. Among abdomen/pelvis metastasis, liver metastasis was more frequently and significantly observed in SQ (22.2% vs 10.4%; *P* = 0.004). However, the frequency of adrenal metastasis or abdomen/pelvis metastasis other than liver/adrenal metastasis showed no statistically significant difference (*P* = 0.442 and *P* = 0.322, respectively). In terms of whole-body metastasis, patients with high metastatic scores (score 3–6) were more frequently found in Adenoca (91.6% vs 73.4%, *P* < 0.001). Among 179 patients with a high total metastatic score, only 15 (8.4%) patients were included in SQ.

**TABLE 3:**
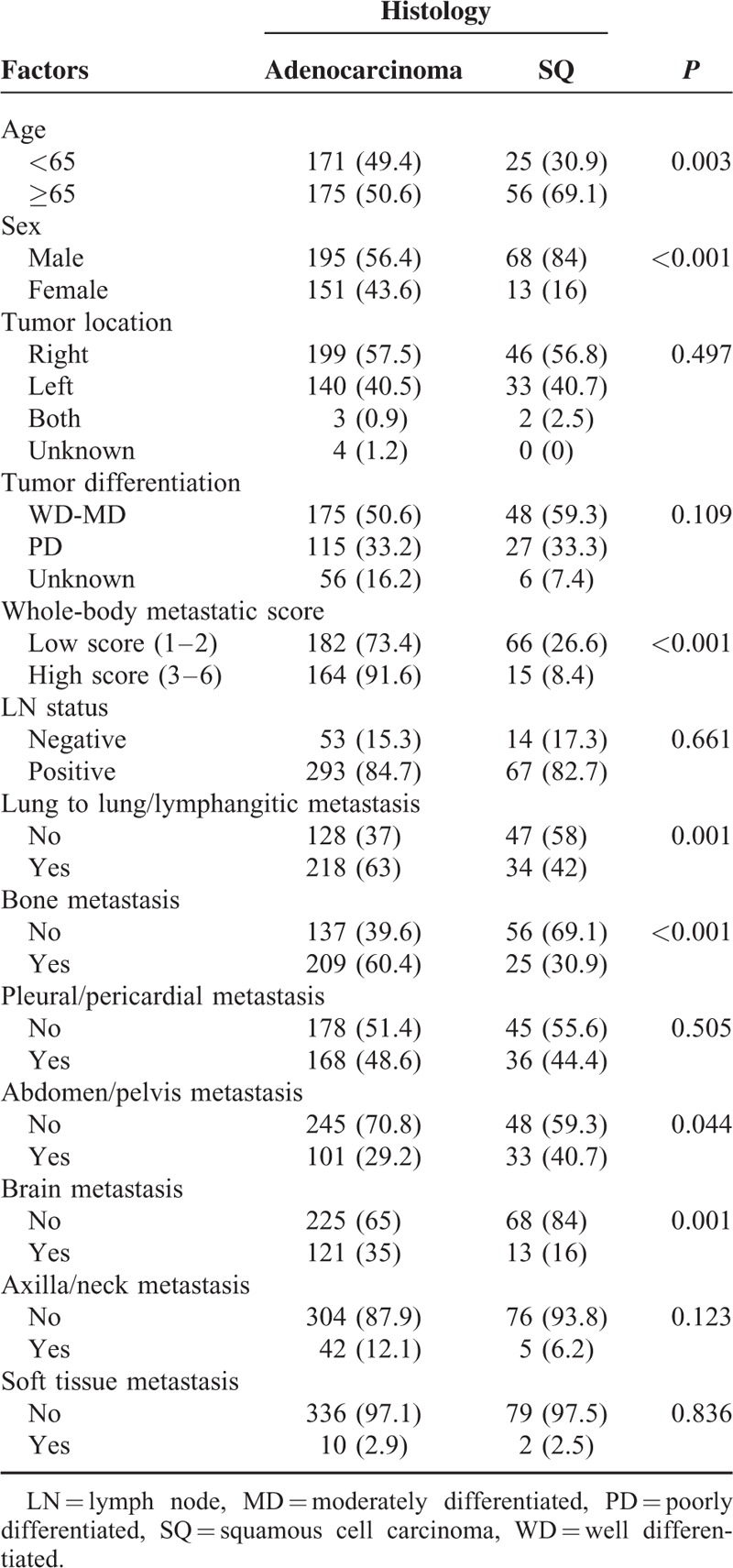
Comparison of Patient, Tumor, and Metastatic Characteristics According to the Histological Subtypes

### Treatment Details

In terms of administered treatment, 232 (67.1%) of 346 Adenoca patients and 41 (50.6%) of 81 SQ patients had received chemotherapy or targeted therapy. The treatment details in 346 Adenoca patients were as follows: only the use of combination chemotherapeutic agents in 88 (25.4%) patients; the use of molecular-targeted agents as first-line treatment in 53 (15.3%) patients; the use of molecular-targeted agents after second-line treatment in 98 (28.3%) patients; and no treatment or lost to follow-up at our institution in 107 (30.9%) patients.

Among 98 patients with activating EGFR mutations, 40 (40.8%) and 28 (28.6%) patients had received targeted therapies as first-line and after second-line treatment, respectively. Ten (10.2%) patients were administered only combination chemotherapeutic agents, and 20 (20.4%) patients did not receive any treatment at our institution.

**FIGURE 1:**
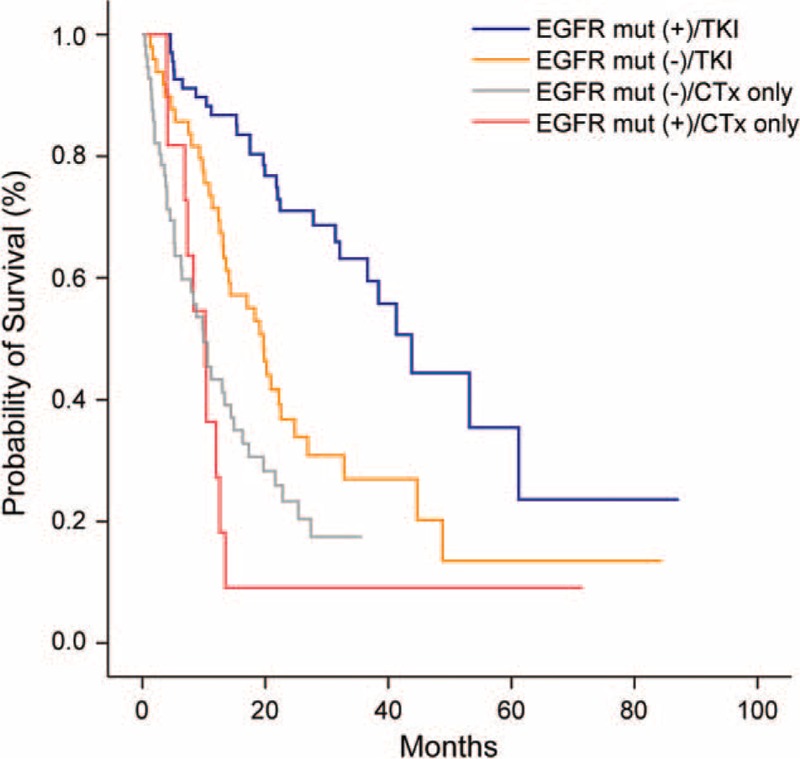
Overall survival curves according to the EGFR mutation status and administered treatment in the Adenoca subgroup with available EGFR mutation statuses and treatment factors (n = 184, *P* < 0.001). Total number of patients in each group was as follows: EGFR mutation (+)/TKI (+) = 68; EGFR mutation (−)/TKI (+) = 49; EGFR mutation (+)/chemotherapy only = 11; and EGFR mutation (−)/chemotherapy only = 56.

Figure 1 shows the OS curves according to the administered treatment in the Adenoca subgroup with available EGFR mutation status that had received chemotherapy or targeted therapy. The number of patients in each group was as follows: (a) EGFR mutation (+)/TKI (+) = 68; (b) EGFR mutation (−)/TKI (+) = 49; (c) EGFR mutation (+)/chemotherapy only = 11; (d) EGFR mutation (−)/chemotherapy only = 56. The median OS values of group (a), (b), (c), and (d) were 43.8 (95% confidence interval [CI] 34.9–52.6), 19.7 (95% CI 15.6–23.8), 10.2 (95% CI 6.9–13.5), and 9.9 (95% CI 6.7–13.1) months, respectively (*P* < 0.001).

### Prognostic Evaluation: Univariate and Multivariate Survival Analyses

**FIGURE 2:**
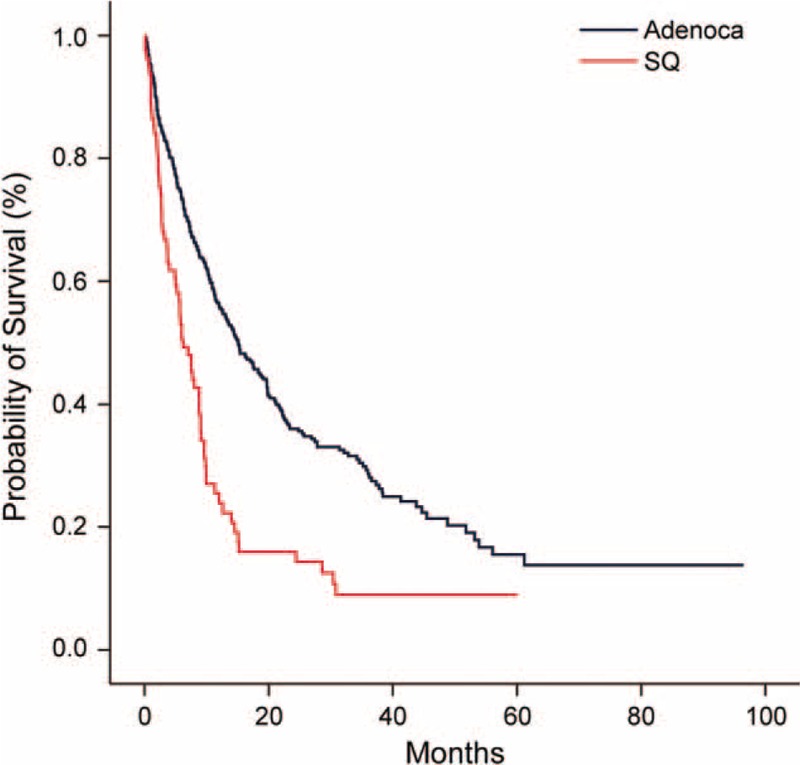
Overall survival curves according to the histological subtype (N = 427: Adenoca = 346 and SQ = 81, *P* < 0.001).

The median follow-up duration was 11.2 months (range, 0.1–96.5 months) at the time of this analysis. During follow-up, a total of 311 (72.8%) patients died. The median estimated OS was 12.1 (95% CI 10.0–14.2) months. Kaplan-Meier analysis demonstrated a statistically significant survival difference according to the histological subtypes (*P* < 0.001), and Figure 2 shows the OS curves according to the histological subtypes. The median OS values in the Adenoca and SQ groups were 15.1 (95% CI 12–18.2) and 6.2 (95% CI 4.4–8.0) months, respectively (*P* < 0.001).

In multivariate analysis for OS, sex (median: 9.0 months in males vs 19.7 months in females; *P* = 0.001), age (median: 19.8 months in patients <65 years vs 7.5 months in patients ≥65 years; *P* < 0.001), histology (median: 15.1 months in the Adenoca group vs 6.2 months in the SQ group; *P* < 0.001), LN status (median: 22 months with no LN metastasis vs 11.4 months with LN metastasis; *P* = 0.032), pleural/pericardial metastasis (median: 13.1 months with no pleural/pericardial metastasis vs 10.9 months with pleural/pericardial metastasis; *P* = 0.003), abdomen/pelvis metastasis (median: 15.3 months with no abdomen/pelvis metastasis vs 8.8 months with abdomen/pelvis metastasis; *P* < 0.001), axilla/neck metastasis (median: 13.1 months with no axilla/neck metastasis vs 8.5 months with axilla/neck metastasis; *P* = 0.006), and treatment factors (median: 18.3 months with chemotherapy/targeted therapy vs 4.7 months with no treatment/loss to follow-up; *P* < 0.001) remained independent prognostic factors. The results of univariate and multivariate analyses are shown in Table 4.

**TABLE 4:**
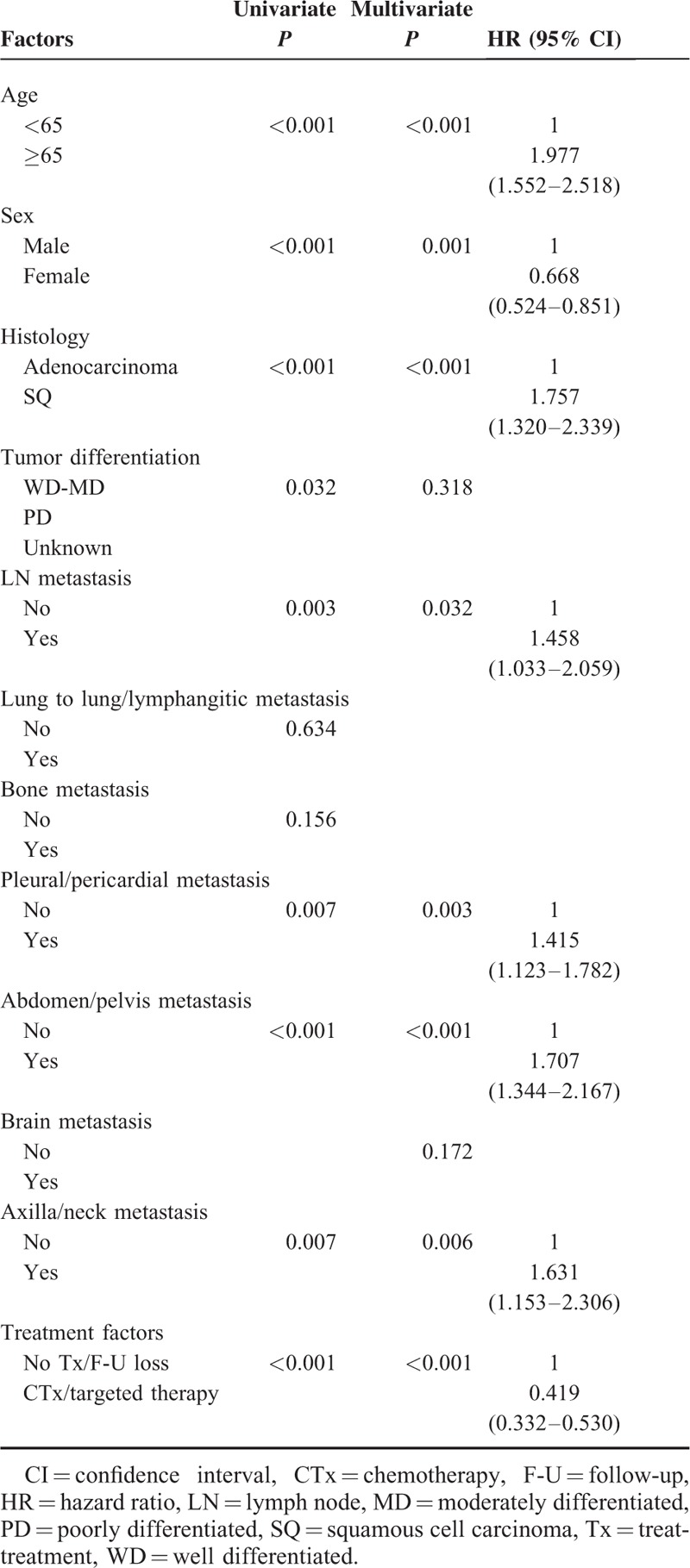
Univariate and Multivariate Analysis for Overall Survival

## DISCUSSION

In the present study, we aimed to compare the patterns of metastases at the initial diagnosis and clinical outcomes according to histological subtype in advanced NSCLC. The study population was 427 patients (346 Adenoca and 81 SQ). In line with the changing epidemiology of lung cancer in the world,^5,18,19^ Adenoca was found as a main histology in our cohort study.

The results of a more frequent distribution of advanced age (≥65 years) and male sex in SQ were concordant with those of previous publications.^4,5,20^ Metastatic distributions were absolutely distinguishing, with higher metastatic scores being found in Adenoca. The metastatic sites were also significantly and differently distributed to the whole body, with tropism for lung, bone, and brain in Adenoca. Although our classification of metastatic scores for whole-body metastatic evaluation had several limitations, the specific organ affinity or a tendency of dissemination to the specific sites might be present. The prevalence of distant metastases in NSCLC has been debated. In some studies,^21,22^ the authors found that patients with Adenoca were at a significantly higher risk for extrathoracic metastases than patients with SQ. Quint et al^23^ reported that brain, bone, liver, and adrenal glands were the most common sites of metastatic disease from NSCLC at presentation. They observed that Adenoca more commonly metastasized to the brain than SQ, and SQ more commonly metastasized to axillary LNs than Adenoca. However, there was no significant difference between Adenoca and SQ for the rates of metastases to bone, lung, or liver.

We evidently observed that although the metastatic potential was much higher in Adenoca, the treatment outcomes were more favorable in Adenoca (Figure 2). These findings suggest that treatment responses associated with well-known molecular alterations^24–30^ or unique biological features^5,13,31^ are more crucial to the clinical outcomes rather than the initial metastatic extent. In this study, 98 (39.4%) among the total 346 Adenoca patients exhibited activating EGFR mutations, and 68 (69.4%) patients had received molecular-targeted agents at our institution. As shown in Figure 1, the OS of the Adenoca subgroup was significantly different based on the EGFR mutation status and type of administered agent. When only chemotherapeutic agents had been employed, the treatment outcomes were poorest, and there was no significant difference in OS according to the EGFR mutation status. However, when molecular-targeted agents had been employed, the OS was significantly better in the group with EGFR mutations than in the group with wild-type EGFR. Even among the population with wild-type EGFR, the use of molecular-targeted agents seemed to lead to superior survival outcomes in our study (Figure 1). Good treatment responses to the molecular-targeted agents regardless of EGFR mutation status are believed to partly contribute to the superior survival results of Adenoca compared with SQ in the present study. It has already been shown that common genetic changes such as EGFR mutations or EML4-ALK translocations strongly affect the prognosis in lung Adenoca.^26–28,32,33^ Because molecular tests for EML4-ALK rearrangements were initiated recently, we did not show the results in this work.

In the multivariate survival analysis, LN metastasis, pleural/pericardial metastasis, abdomen/pelvis metastasis, and axilla/neck metastasis, as determined by 18F-FDG-PET/CT, as well as traditional prognostic factors such as age, sex, and histological subtype, were independent prognosticators for OS. Meaningful factors in our study might be suggested as unique and novel prognosticators in metastatic NSCLC.

Our recent publication^13^ suggested that increased serum carcinoembryonic antigen (CEA) levels were significantly associated with whole-body metastasis, particularly to the bone, brain, and lung to lung/lymphangitic spread. Multivariate analyses have shown that the odds ratios for bone and brain metastasis were 2.549 (95% CI 1.606–4.047) and 2.098 (95% CI 1.251–3.52), respectively. It has been evidently recognized that elevated serum CEA levels are significantly related to Adenoca histology. Our comparative results discovered that lung to lung/lymphangitic spread, bone metastasis, and brain metastasis, which were significantly correlated with increased serum CEA levels in previous work, were also significantly and more frequently distributed in Adenoca compared with SQ in the current study. CEA was originally characterized as an adhesive molecule and a chemoattractant.^34^ Many preclinical and experimental studies disclosed the promoting adhesive function of tumor cells to the endothelium activated by CEA.^35^ Thus, serum CEA may partly contribute to the metastatic distribution into the whole-body and has a strong affinity to specific sites by its function as an adhesion molecule.

The following limitations could be found in our study. Metastatic evaluation was performed using clinical metabolic imaging, not pathological confirmation. Utilization of metabolic imaging has several limitations such as a high false-positive rate in mediastinal LN staging, particularly in SQ histology.^36,37^ Treatment characteristics were heterogeneous among patients, and 63.9% (n = 273) of the total population had finally received any type of treatment at our institution. Nonetheless, we discovered distinctive patterns of primary metastases according to the different lung cancer histological subtypes.

In summary, we observed unique patterns of initial metastases and clinical outcomes according to histological subtype in stage IV NSCLC. The underlying mechanism of these distinctive metastatic characteristics and more detailed features of different tumor biologies need to be resolved in future studies.
