# Distinctive Patterns of Initially Presenting Metastases and Clinical Outcomes According to the Histological Subtypes in Stage IV Non-Small Cell Lung Cancer: Erratum

**DOI:** 10.1097/01.md.0000482790.51005.5b

**Published:** 2016-04-18

In the article “Distinctive Patterns of Initially Presenting Metastases and Clinical Outcomes According to the Histological Subtypes in Stage IV Non-Small Cell Lung Cancer”,^1^ which appeared in Volume 95, Issue 6 of *Medicine*, the authors’ full names were not originally given in the byline. The article has since been corrected online.
